# Durability of Humoral Responses after the Second Dose of mRNA BNT162b2 Vaccine in Residents of a Long Term Care Facility

**DOI:** 10.3390/vaccines10030446

**Published:** 2022-03-14

**Authors:** Alessia Lai, Barbara Caimi, Marco Franzetti, Annalisa Bergna, Rossella Velleca, Antonella Gatti, Pier Luigi Rossi, Marco D’Orso, Fabrizio Pregliasco, Claudia Balotta, Giuseppe Calicchio

**Affiliations:** 1 Department of Biomedical and Clinical Sciences L. Sacco, University of Milan, 20157 Milan, Italy; annalisa.bergna@unimi.it (A.B.); claudia.balotta@unimi.it (C.B.); 2 Azienda Servizi alla Persona, Istituti Milanesi Martinitt e Stelline e Pio Albergo Trivulzio, 20146 Milan, Italy; barbara.caimi@pioalbergotrivulzio.it (B.C.); rossella.velleca@pioalbergotrivulzio.it (R.V.); antonella.gatti@pioalbergotrivulzio.it (A.G.); dip.sociosanitario@pioalbergotrivulzio.it (P.L.R.); direzione.generale@pioalbergotrivulzio.it (G.C.); 3 Infectious Diseases Unit, Legnano General Hospital, ASST Ovest Milanese, 20025 Legnano, Italy; franzetti.marco@gmail.com; 4 Department of Medicine and Surgery, University of Milan-Bicocca, 20900 Monza, Italy; marco.dorso@synlab.it; 5 Department of Biomedical Sciences, IRCCS Istituto Ortopedico Galeazzi, University of Milan, 20161 Milan, Italy; fabrizio.pregliasco@unimi.it

**Keywords:** long-term care facilities, humoral responses, nucleocapsid serology, COVID-19, vaccines

## Abstract

Residents of long-term care facilities (LTCFs) have been dramatically hit by the COVID-19 pandemic on a global scale as older age and comorbidities pose an increased risk of severe disease and death. The aim of the study was to assess the quantity and durability of specific antibody responses to SARS-CoV-2 after the first cycle (two doses) of BNT162b2 vaccine. To achieve this, SARS-CoV-2 Spike-specific IgG (S-IgG) titers was evaluated in 432 residents of the largest Italian LTCF at months 2 and 6 after vaccination. By stratifying levels of humoral responses as high, medium, low and null, we did not find any difference when comparing the two time points; however, the median levels of antibodies halved overtime. As positive nucleocapsid serology was associated with a reduced risk of a suboptimal response at both time points, we conducted separate analyses accordingly. In subjects with positive serology, the median level of anti-S IgG slightly increased at the second time point, while a significant reduction was observed in patients without previous exposure to the virus. At month 6, diabetes alone was associated with an increased risk of impaired response. Our data provide additional insights into the longitudinal dynamics of the immune response to BNT162b2 vaccination in the elderly, highlighting the need for SARS-CoV-2 antibody monitoring following third-dose administration.

## 1. Introduction

After the emergence of SARS-CoV-2 (Severe Acute Respiratory Syndrome Coronavirus 2), the etiologic agent of the Coronavirus Disease 19 (COVID-19) in December 2019, its global spread had caused more than 151 million cases and 1.7 million deaths by mid-February in Europe (https://www.ecdc.europa.eu/en/geographical-distribution-2019-ncov-cases accessed on 15 February 2022).

Italian data confirmed the association between older patient populations and highest mortality. During the first wave of the pandemic in Italy, the overall mortality rate was 7.2%, with about 40% of cases accounting for patients aged 70 and above (Istituto Superiore di Sanità, ISS,) [1,2]. Risk factors for severe COVID-19 included concurrent chronic diseases typical in older age. Among the illness conditions that frequently occur in COVID-19 patients, those most common include high blood pressure, cardiovascular diseases, chronic obstructive pulmonary disease, diabetes mellitus and malignancy. Research findings indicated that the risk of death is significantly higher in patients with these concurrent diseases than in those who have not comorbidities. Moreover, old age increases the risk of severe COVID-19 clinical course and death [3,4]. Additional factors worsening the COVID-19 emergency within geriatric population may include intrinsic multidimensional features characterizing older subjects, such as disabilities, cognitive and mood disorders, polypharmacotherapy, social isolation, nutritional deficits and extrinsic factors detectable in widespread ageism, the generalized lack of geriatric culture and alleged errors in the management of long-term care facilities [5].

The vulnerability of long-term care facilities (LTCFs) to respiratory disease outbreaks, including influenza and other commonly circulating human coronaviruses such as the common cold, is well recognized [6,7] and the spread of COVID-19 reflected the same frailty. Although LTCFs have been disproportionately affected by the COVID-19 pandemic and high rates of infection and deaths have reported [8,9], survivors developed high SARS-CoV-2 antibody levels, including neutralising antibodies, after infection [10,11,12,13]. However, whether prior SARS-CoV-2 infection influences a higher response to vaccine and protects against re-infection is not firmly established in old individuals. While current COVID-19 vaccines appear to be effective in reducing severe illness and death, information regarding antibody response to COVID-19 vaccines is limited in old age at present [14,15].

Therefore, the objective of this study was to quantify the magnitude and longevity of antibody responses in vaccinated older residents of the largest Italian LTCF with and without prior COVID-19 infection six months after the second dose of mRNA BNT162b2 vaccine.

## 2. Materials and Methods

### 2.1. Study Population

Four hundred and thirty-two residents of the LTCF Pio Albergo Trivulzio were studied. First-dose administration of BNT162b2 vaccine began on 27th December 2020 and occurred until 31 January 2021. The second dose was administered 21 days after and was completed on 25 February 2021.

All residents provided blood samples at 2 (median 64 days, Inter-Quartile Range, IQR: 63–65 days) and 6 months (median 202 days, IQR: 199–203 days) after the second dose of the BNT162b2 mRNA vaccine.

At first time point, samples were concomitantly screened for SARS-CoV-2 nucleocapsid-specific IgG (N-IgG) and SARS-CoV-2 Spike-specific IgG (S-IgG) antibodies, while at the second time point only S-IgG antibodies were measured as, in the time interval between the two samples, no infection cases were reported.

All participants provided written informed consent. If residents lacked the capacity to consent, a personal or nominated consultee was identified to act on their behalf. All data used in this study were previously anonymized as required by the Italian Data Protection Code (Legislative Decree 196/2003) and the general authorizations issued by the Data Protection Authority. Ethics Committee approval was deemed unnecessary because, under Italian law, it is only required in the case of prospective clinical trials of medical products for clinical use (Art. 6 and Art. 9 of Legislative Decree 211/2003). The study was conducted in compliance with Good Clinical Practice (https://ichgcp.net/it accessed on 15 February 2022) and the declaration of Helsinki.

### 2.2. SARS-CoV-2 Antibody Assays

The presence and magnitude of antibody response in this population was determined using Elecsys Anti-SARS-CoV-2 S and N (Roche, Basel, Swiss).

Response to vaccination in residents, estimated as total antibodies to SARS-CoV-2 spike protein receptor binding domain (RBD), was arbitrarily classified by stratifying levels of anti-S IgG values in 4 levels: >1000, 101–1000, 1–100 and <1 BAU/mL (Binding Antibody Unit/mL), defined as high, medium, low and null response, respectively, as already published [16]. The degree of response to vaccination at different time points was assessed considering the four previously defined levels; the suboptimal response was defined as low and null compared to the high and medium response, while null response was considered as levels defined otherwise.

### 2.3. Statistical Analysis

Descriptive analyses of demographic and clinical data are presented as median and Inter-Quartile Range when continuous and as frequency and proportion (%) when categorical. To compare normally distributed, non-normally distributed continuous, and categorical variables, parametric tests (t test and ANOVA), nonparametric tests (Mann–Whitney and Kruskal–Wallis) and the Pearson χ2 test (or Fisher exact test, when necessary) were used, respectively. The primary endpoint was the risk of inadequate response to vaccine (anti-S IgG values < 100 BAU/mL), evaluated by means of a logistic regression model, also correcting for gender, age, comorbidities and immune modulatory treatments. Separate analyses were conducted for patients who tested positive or negative for anti-nucleocapsid serology. Significance was established at *p <* 0.05. A data analysis was performed using the IBM SPSS Statistics version 25.

## 3. Results

Among LTCF residents 81.7% (*n* = 353) were female and the median age was 88 years (IQR: 82–92).

According to the level of response at the first time point (two months after two doses of vaccine), high, medium, low and null titers were present in 63% (*n* = 272), 21.8% (*n* = 94), 11.8% (*n* = 51) and 3.5% (*n* = 15) of cases, respectively. No differences were detected at the second time point and the proportion of titers were, respectively, as follows: 60.6% (*n* = 262), 22.5% (*n* = 97), 16% (*n* = 69) and 0.9% (*n* = 4).

Eleven residents (2.5%) showed an increase in their humoral response at the second time point as null responders became low responders. These individuals, of which eight (72.7%) were over 90 years of age, did not have a previous clinical diagnosis of COVID-19 or a positive serology, and nine (81.8%) of these cases did not use corticosteroids.

A significant decrease in the median level of anti-S IgG was observed between the two time points (*p* < 0.001) with a median of 7500 BAU/mL, corresponding to the upper limit of quantification of the assay (IQR: 848- > 7500 BAU/mL), and 2955 (IQR: 190- > 7500 BAU/mL) at the first and second time point, respectively.

The SARS-CoV-2 anti-nucleocapsid antibodies were positive in 254 cases (58.8%), even though clinical signs of infection in the past were reported only in one third (29.4%) of the total subjects (*n* = 127).

Considering all levels of serological response, at month two, a significantly higher response was found in residents with a previous clinical diagnosis of COVID-19 and in those with positive nucleocapsid serology. In detail, among these subjects, we did not find any case of a null response to vaccine. Regarding patients with a previous clinical diagnosis, the proportion of low responders was 5.9% (3/51) and 2.9% (2/69) at the first and second time points, respectively. An equal proportion was observed in subjects with positive nucleocapsid serology.

**Figure 1:**
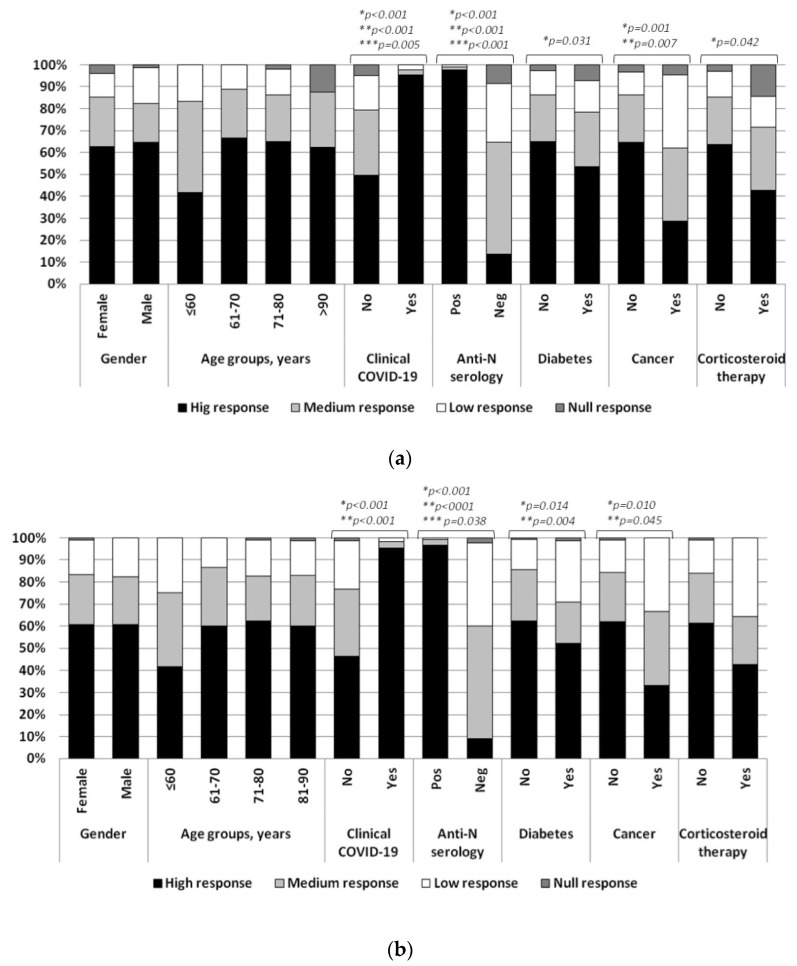
(**a**) Grade of response to SARS-CoV-2 vaccine at 1st and (**b**) at 2nd time points. * Overall response (high, medium, low and null); ** Suboptimal response (high and medium vs. low and null); *** Null response (high, medium and low vs. null).

In contrast, corticosteroid administration and diabetes reduced all levels of specific antibodies. However, significant associations were observed only when considering all levels of response. Residents with neoplastic disease showed a significant difference in the distribution of the IgG–S titers considering all levels and the suboptimal response, but not the null response (38.1% vs. 13.9%) (Figure 1a).

Regarding the degree of response to vaccination at month six, a significant difference was observed in the distribution of antibody responses in patients with diabetes and neoplastic disease considering all the levels and suboptimal levels of response (29% vs. 14.4% and 33.3% vs. 15.9% for diabetes and neoplastic disease, respectively), but not for null response (1.4% vs. 0.8% and 0% vs. 1% for diabetes and neoplastic disease, respectively). According to the results at first time point, residents with positive nucleocapsid serology showed a significantly higher response considering all levels of serologic responses. Higher levels were also present in subjects with a previous clinical diagnosis of COVID-19. Differently from the first time point, no association was observed with respect to corticosteroid administration (Figure 1b). At both times, no associations were detected for gender and age groups. At the first time point, 11 out of 15 null responders (73.3%) were older than 80 years and 44/51 (86.3%) subjects with low response were older than 71 years; at the second time point all null responders (*n* = 4) were older than 71 years while no difference was observed in the proportion of low-responders (59/69, 85.5%).

Moreover, in patients with ischemic heart diseases, the degree of response to vaccination showed a significant difference considering all the levels of specific antibodies. Ischemic heart disease did not influence a null response, as null responders without such clinical condition were prevalent at both time points (at month 2: 93.3%,14/15, *p* = 0.038; at month 6: 100%, 4/4, *p* = 0.026). No association was observed for patients affected by chronic lung diseases.

Independent predictors of an increased risk of suboptimal response in the multivariate analysis at month 2 was only the advanced age. Positive nucleocapsid serology was strongly associated with a reduced risk of a suboptimal response to vaccination at both time points. At month 6, diabetes mellitus was associated with an increased risk (Table 1).

Separate analyses were therefore promptly conducted for residents according to results of SARS-CoV-2 anti-nucleocapsid antibodies. In subjects with positive serology, the median level of anti-S IgG slightly increased at the second time point even if this difference was not significant (7139 vs. 7401 BAU/mL). On the contrary, a significant difference was observed in patients without previous exposure to the virus, where median titers nearly halved in the study period (793 vs. 483, *p* = 0.002). A weak association was observed for all levels of response and age (*p* = 0.046), with 10/15 null responders (66.7%) in patients aged >80 years when evaluated at the second time point.

In the former group, a multivariate analysis at the first time point indicated an increased risk of suboptimal response for cancer (*p* = 0.05; OR: 23.92; 95%CI: 0.90–63.8), corticosteroid therapy (*p* = 0.049; OR: 30.67; 95%CI: 1.01–92.8) and age >80 years (*p* = 0.038; OR: 0.05; 95%CI: 0.003–0.84). No significant association were observed at second time point.

In the latter group, advanced age (*p* = 0.010; OR: 2.4; 95%CI: 1.23–4.66) and diabetes mellitus (*p* = 0.008; OR: 3.43; 95%CI: 1.37–8.56) were associated with an increased risk of a suboptimal response at the first and second time point, respectively.

## 4. Discussion

Neutralizing antibodies have been demonstrated to be the strongest correlate of protection [17,18]. Nevertheless, neutralizing assays are complex and time-consuming and anti-S IgG titers are commonly used as surrogate marker to understand efficacy and durability of vaccine administration [19,20,21]. A recent paper reported a consistent correlation between these parameters, indicating that specific antibody responses to SARS-CoV-2 are time-dependent [22]. Six months after the first cycle of vaccination with BNT162b2, the immune humoral responses waned especially in men, individuals older than 65 years and among individuals with immunosuppression [23].

Although the thresholds for positivity and cut-off values of the SARS-CoV-2 antibody provided by different assays differ, and their diagnostic value has not yet been established and standardized, its quantification after vaccination is highly relevant in identifying possible vaccine failure and estimating the level and duration of protection [24].

The theme of determining the necessary amount of antibodies according to a certain level of immunity is currently being studied and debated extensively. A recent study [25] compared the concentrations of binding antibodies before infections with SARS-CoV-2 Delta or Omicron variants. Each individual infected with the Omicron variant was compared by age, sex and vaccination status with a person infected with the Delta variant with known antibody titers from 15 days to 2 months before infection. The authors stated that infections with the SARS-CoV-2 Omicron variant can occur despite high concentrations of antibodies, even at concentrations 2.4 higher than infections with the Delta variant (6967 vs. 2905 BAU/mL). A previous study [26] suggested that anti-S antibody titers above 141 BAU/mL were related to the presence of neutralizing antibodies against wild-type and Alpha SARS-CoV-2 variant. These papers supported the stratification of anti-S IgG values in different levels to study the correlates of infection. During the study period, no infections occurred in LTCF residents according to the high amount of antibodies detected. However, at month 6, patients without previous exposure to SARS-CoV-2 infection showed very low titers, possibly conferring a certain level of protection against the circulating variants at a time period before the introduction of Delta in Italy.

We previously reported [16] a substantially optimal response to vaccine after 2 months from the second dose administration in subjects living in LTCF, with some limitations in those affected by comorbidities and treated with corticosteroids. However, time-dependent variations are expected in the immunological response to vaccine, which may specifically affect the elderly population [27]. Therefore, we further analyzed old-aged residents of the same LTCF at six months after the second dose of vaccine. A residual number of subjects (11 patients) showed a weak increase in specific antibody levels and the proportions of responsive residents remained high at the second time point (84.7% vs. 83.1%). Notably, median titers of anti-S IgG were considerably lower in the entire population, indicating a fast decay of response, which is in agreement with previous reports [18,28,29]. Overall, our studies confirm the accumulating evidence [14,30,31,32] that long term humoral response and vaccine effectiveness in previously infected persons were superior to that in recipients who did not experience COVID-19 and received two doses of the vaccine [33]. By analyzing residents with or without previous COVID-19 infection, we demonstrated that median levels of specific antibody levels significantly decreased in the latter population over time, displaying titer values at least 10 times lower compared to the formers.

At the second time point of the study, no predictors of an increased risk of suboptimal response to vaccine were observed in patients who experienced COVID-19 disease. We previously reported that an older age in LTCF residents was strongly associated with an increased risk of a null response to vaccine, hypothesizing that immunosenescence, which reduced adaptive immune responses, could be responsible for this finding [16]. When studying the multivariate results of the second time point, these data were not confirmed, probably because a small fraction of non-responder residents at month 2 showed a certain specific reactivity at month 6. This finding is in agreement with Collier et al. [34], indicating that the age-related immune response may be heterogeneous.

Similarly, age was not an independent predictor when stratifying the hosts according to anti-nucleocapsid serology.

Interestingly, at the second time point, the comorbidity of diabetes mellitus alone was associated with an increased risk of an impaired response to the vaccine, specifically for those who did not experience SARS-CoV-2 infection. According to the literature data, patients with both type 1 and 2 diabetes have an increased risk of a more severe course of COVID-19 [35,36]. As diabetes represents an independent risk for disease severity, subjects living with diabetes were included among priority groups for vaccination programmes. Some studies have reported a lower humoral response in diabetic patients after two doses of BNT162b2 [37,38], even if they did not experience a previous exposure to COVID-19.

Our study has some limitations as we did not include a control group of subjects. The male population was underrepresented and conclusions on gender differences in vaccine response need further studies and require a larger population. Furthermore, our results are limited to antibody responses after the second dose without considering the effects of a booster dose.

The present study did not assess cell-mediated immunity; however, our data suggest the importance of monitoring the total antibody concentrations as a surrogate marker to optimize vaccination strategies. A reliable estimation of the duration and degree of protection provided by vaccines is a compelling issue in the fragile population and specifically in elderly persons. Our data provide additional insights into the longitudinal dynamics of humoral immune response to BNT162b2 vaccination in old age. The importance of determining immune correlates of protection after vaccination remains a turning point of intervention strategies. Strategies to prolong host immunity need to be evaluated in order to protect the population against SARS-CoV-2 and its variants. Overall, our data support the need for booster doses in the elderly, as additional vaccination provides a method by which to control transmission together with the maintenance of stringent measures of prevention in LTCFs. Future studies will help to determine the longer-term effectiveness of the booster dose against current and emerging variants in the elderly by studying both humoral and cellular responses to SARS-CoV-2 to understand the type and function of antibodies produced after vaccination, the neutralizing capacity of the antibodies and the persistence of their protective effects.

## Figures and Tables

**Table 1:** Multivariable logistic regression analyses of the risk of suboptimal response to SARS-CoV-2 vaccination after 2 and 6 months in residents of a long-term care facility.

	1st Time Point			2nd Time Point		
	*p* Value	OR *	95% CI **	*p* Value	OR	95% CI
Age, per 10 year higher	0.007	2.460	1.13–6.14	0.136	2.630	1.13–6.14
Gender, females vs. males	0.264	0.571	0.21–1.53	0.722	0.834	0.31–2.27
Positive N serology	0.000	0.016	0.004–0.067	0.000	0.007	0.001–0.049
Diabetes mellitus	0.426	1.431	0.59–3.46	0.013	3.080	1.27–7.48
Cancer	0.231	2.198	0.61–7.98	0.835	0.869	0.23–3.26
Corticosteroid therapy	0.252	2.394	0.54–10.67	0.142	3.384	0.67–17.26

* OR: Odds Ratio. ** 95%CI: 95% Confidence Interval.

## Data Availability

All the data have been reported in the manuscript.

## References

[1] De Girolamo G., Bellelli G., Bianchetti A., Starace F., Zanetti O., Zarbo C., Micciolo R. (2020). Older People Living in Long-Term Care Facilities and Mortality Rates During the COVID-19 Pandemic in Italy: Preliminary Epidemiological Data and Lessons to Learn. Front. Psychiatry.

[2] Dorrucci M., Minelli G., Boros S., Manno V., Prati S., Battaglini M., Corsetti G., Andrianou X., Riccardo F., Fabiani M. (2021). Excess Mortality in Italy During the COVID-19 Pandemic: Assessing the Differences Between the First and the Second Wave, Year 2020. Front. Public Health.

[3] Lynch S.M., Guo G., Gibson D.S., Bjourson A.J., Rai T.S. (2021). Role of Senescence and Aging in SARS-CoV-2 Infection and COVID-19 Disease. Cells.

[4] Romero Starke K., Reissig D., Petereit-Haack G., Schmauder S., Nienhaus A., Seidler A. (2021). The isolated effect of age on the risk of COVID-19 severe outcomes: A systematic review with meta-analysis. BMJ Glob. Health.

[5] Bencivenga L., Rengo G., Varricchi G. (2020). Elderly at time of COronaVIrus disease 2019 (COVID-19): Possible role of immunosenescence and malnutrition. GeroScience.

[6] Hand J., Rose E.B., Salinas A., Lu X., Sakthivel S.K., Schneider E., Watson J.T. (2018). Severe Respiratory Illness Outbreak Associated with Human Coronavirus NL63 in a Long-Term Care Facility. Emerg. Infect. Dis..

[7] Lansbury L.E., Brown C.S., Nguyen-Van-Tam J.S. (2017). Influenza in long-term care facilities. Influenza Other Respir. Viruses.

[8] McMichael T.M., Currie D.W., Clark S., Pogosjans S., Kay M., Schwartz N.G., Lewis J., Baer A., Kawakami V., Lukoff M.D. (2020). Epidemiology of Covid-19 in a Long-Term Care Facility in King County, Washington. N. Engl. J. Med..

[9] Patel M.C., Chaisson L.H., Borgetti S., Burdsall D., Chugh R.K., Hoff C.R., Murphy E.B., Murskyj E.A., Wilson S., Ramos J. (2020). Asymptomatic SARS-CoV-2 Infection and COVID-19 Mortality During an Outbreak Investigation in a Skilled Nursing Facility. Clin. Infect. Dis. Off. Publ. Infect. Dis. Soc. Am..

[10] Ladhani S.N., Jeffery-Smith A., Patel M., Janarthanan R., Fok J., Crawley-Boevey E., Vusirikala A., Fernandez Ruiz De Olano E., Perez M.S., Tang S. (2020). High prevalence of SARS-CoV-2 antibodies in care homes affected by COVID-19: Prospective cohort study, England. EClinicalMedicine.

[11] Wajnberg A., Mansour M., Leven E., Bouvier N.M., Patel G., Firpo-Betancourt A., Mendu R., Jhang J., Arinsburg S., Gitman M. (2020). Humoral response and PCR positivity in patients with COVID-19 in the New York City region, USA: An observational study. Lancet Microbe.

[12] Deeks J.J., Dinnes J., Takwoingi Y., Davenport C., Spijker R., Taylor-Phillips S., Adriano A., Beese S., Dretzke J., Ferrante di Ruffano L. (2020). Antibody tests for identification of current and past infection with SARS-CoV-2. Cochrane Database Syst. Rev..

[13] Graham N.S.N., Junghans C., McLaren R., Randell P., Lang N., Ladhani S.N., Sharp D.J., Sanderson F. (2021). High rates of SARS-CoV-2 seropositivity in nursing home residents. J. Infect..

[14] Hall V.J., Foulkes S., Charlett A., Atti A., Monk E.J.M., Simmons R., Wellington E., Cole M.J., Saei A., Oguti B. (2021). SARS-CoV-2 infection rates of antibody-positive compared with antibody-negative health-care workers in England: A large, multicentre, prospective cohort study (SIREN). Lancet.

[15] Nace D.A., Kip K.E., Mellors J.W., Peck Palmer O.M., Shurin M.R., Mulvey K., Crandall M., Sobolewski M.D., Enick P.N., McCormick K.D. (2021). Antibody Responses After mRNA-Based COVID-19 Vaccination in Residential Older Adults: Implications for Reopening. J. Am. Med. Dir. Assoc..

[16] Caimi B., Franzetti M., Velleca R., Lai A., Gatti A., Luigi R., D’Orso M., Pregliasco F., Balotta C., Calicchio G. (2022). Sero-Survey on Long-Term Care Facility Residents Reveals Increased Risk of Sub-Optimal Antibody Response to BNT162B2: Implications for Breakthrough Prevention. BMC Geriatr..

[17] Bergwerk M., Gonen T., Lustig Y., Amit S., Lipsitch M., Cohen C., Mandelboim M., Levin E.G., Rubin C., Indenbaum V. (2021). Covid-19 Breakthrough Infections in Vaccinated Health Care Workers. N. Engl. J. Med..

[18] Khoury D.S., Cromer D., Reynaldi A., Schlub T.E., Wheatley A.K., Juno J.A., Subbarao K., Kent S.J., Triccas J.A., Davenport M.P. (2021). Neutralizing antibody levels are highly predictive of immune protection from symptomatic SARS-CoV-2 infection. Nat. Med..

[19] Boccuto A., Dragoni F., Bergna A., Ventura C.D., Giammarino F., Saladini F., Pezzati L., Zehender G., Zazzi M., Vicenti I. (2022). Decreased neutralization of the Eta SARS-CoV-2 variant by sera of previously infected and uninfected vaccinated individuals. J. Infect..

[20] Dolscheid-Pommerich R., Bartok E., Renn M., Kümmerer B.M., Schulte B., Schmithausen R.M., Stoffel-Wagner B., Streeck H., Saschenbrecker S., Steinhagen K. (2022). Correlation between a quantitative anti-SARS-CoV-2 IgG ELISA and neutralization activity. J. Med. Virol..

[21] Demonbreun A.R., Sancilio A., Velez M.P., Ryan D.T., Saber R., Vaught L.A., Reiser N.L., Hsieh R.R., D’Aquila R.T., Mustanski B. (2021). Comparison of IgG and neutralizing antibody responses after one or two doses of COVID-19 mRNA vaccine in previously infected and uninfected individuals. EClinicalMedicine.

[22] Đaković Rode O., Bodulić K., Zember S., Cetinić Balent N., Novokmet A., Čulo M., Rašić Ž., Mikulić R., Markotić A. (2022). Decline of Anti-SARS-CoV-2 IgG Antibody Levels 6 Months after Complete BNT162b2 Vaccination in Healthcare Workers to Levels Observed Following the First Vaccine Dose. Vaccines.

[23] Levin E.G., Lustig Y., Cohen C., Fluss R., Indenbaum V., Amit S., Doolman R., Asraf K., Mendelson E., Ziv A. (2021). Waning Immune Humoral Response to BNT162b2 Covid-19 Vaccine over 6 Months. N. Engl. J. Med..

[24] Infantino M., Pieri M., Nuccetelli M., Grossi V., Lari B., Tomassetti F., Calugi G., Pancani S., Benucci M., Casprini P. (2021). The WHO International Standard for COVID-19 serological tests: Towards harmonization of anti-spike assays. Int. Immunopharmacol..

[25] Dimeglio C., Migueres M., Mansuy J.M., Saivin S., Miedougé M., Chapuy-Regaud S., Izopet J. (2022). Antibody titers and breakthrough infections with Omicron SARS-CoV-2. J. Infect..

[26] Dimeglio C., Herin F., Martin-Blondel G., Miedougé M., Izopet J. (2022). Antibody titers and protection against a SARS-CoV-2 infection. J. Infect..

[27] Ciabattini A., Nardini C., Santoro F., Garagnani P., Franceschi C., Medaglini D. (2018). Vaccination in the elderly: The challenge of immune changes with aging. Semin. Immunol..

[28] Salvagno G.L., Henry B.M., Pighi L., De Nitto S., Gianfilippi G., Lippi G. (2022). The pronounced decline of anti-SARS-CoV-2 spike trimeric IgG and RBD IgG in baseline seronegative individuals six months after BNT162b2 vaccination is consistent with the need for vaccine boosters. Clin. Chem. Lab. Med..

[29] Naaber P., Tserel L., Kangro K., Sepp E., Jürjenson V., Adamson A., Haljasmägi L., Rumm A.P., Maruste R., Kärner J. (2021). Dynamics of antibody response to BNT162b2 vaccine after six months: A longitudinal prospective study. Lancet Reg. Health. Eur..

[30] Dan J.M., Mateus J., Kato Y., Hastie K.M., Yu E.D., Faliti C.E., Grifoni A., Ramirez S.I., Haupt S., Frazier A. (2021). Immunological memory to SARS-CoV-2 assessed for up to 8 months after infection. Science.

[31] Vanshylla K., Di Cristanziano V., Kleipass F., Dewald F., Schommers P., Gieselmann L., Gruell H., Schlotz M., Ercanoglu M.S., Stumpf R. (2021). Kinetics and correlates of the neutralizing antibody response to SARS-CoV-2 infection in humans. Cell Host Microbe.

[32] Anderson M., Stec M., Rewane A., Landay A., Cloherty G., Moy J. (2021). SARS-CoV-2 Antibody Responses in Infection-Naive or Previously Infected Individuals After 1 and 2 Doses of the BNT162b2 Vaccine. JAMA Netw. Open.

[33] Tarkowski M., de Jager W., Schiuma M., Covizzi A., Lai A., Gabrieli A., Corbellino M., Bergna A., Ventura C.D., Galli M. (2021). Anti-SARS-CoV-2 Immunoglobulin Isotypes, and Neutralization Activity Against Viral Variants, According to BNT162b2-Vaccination and Infection History. Front. Immunol..

[34] Collier D.A., Ferreira I.A.T.M., Kotagiri P., Datir R.P., Lim E.Y., Touizer E., Meng B., Abdullahi A., Baker S., Dougan G. (2021). Age-related immune response heterogeneity to SARS-CoV-2 vaccine BNT162b2. Nature.

[35] Barron E., Bakhai C., Kar P., Weaver A., Bradley D., Ismail H., Knighton P., Holman N., Khunti K., Sattar N. (2020). Associations of type 1 and type 2 diabetes with COVID-19-related mortality in England: A whole-population study. Lancet Diabetes Endocrinol..

[36] McGurnaghan S.J., Weir A., Bishop J., Kennedy S., Blackbourn L.A.K., McAllister D.A., Hutchinson S., Caparrotta T.M., Mellor J., Jeyam A. (2021). Risks of and risk factors for COVID-19 disease in people with diabetes: A cohort study of the total population of Scotland. Lancet Diabetes Endocrinol..

[37] Yelin I., Katz R., Herzel E., Berman-Zilberstein T., Ben-Tov A., Kuint J., Gazit S., Patalon T., Chodick G., Kishony R. (2021). Associations of the BNT162b2 COVID-19 vaccine effectiveness with patient age and comorbidities. medRxiv.

[38] Ali H., Alterki A., Sindhu S., Alahmad B., Hammad M., Al-Sabah S., Alghounaim M., Jamal M.H., Aldei A., Mairza M.J. (2021). Robust Antibody Levels in Both Diabetic and Non-Diabetic Individuals After BNT162b2 mRNA COVID-19 Vaccination. Front. Immunol..

